# Gut-Thyroid axis: How gut microbial dysbiosis associated with euthyroid thyroid cancer

**DOI:** 10.7150/jca.66816

**Published:** 2022-03-28

**Authors:** Hafiz Muhammad Ishaq, Imran Shair Mohammad, Riaz Hussain, Rashida Parveen, Jafir Hussain Shirazi, Yang Fan, Muhammad Shahzad, Khezar Hayat, Huan Li, Ayesha Ihsan, Kiran Sher Muhammad, Muhammad Usman, Siruo Zhang, Lu Yuan, Shakir Ullah, Ana Cláudia Paiva-Santos, Jiru Xu

**Affiliations:** 1 Department of Microbiology and Immunology, Key Laboratory of Environment and Genes Related to Diseases of Chinese Ministry of Education, School of Medicine, Xi'an Jiaotong University, Xi'an, China; 2 Faculty of Veterinary and Animal Sciences, Muhammad Nawaz Shareef University of Agriculture Multan 66000, Pakistan; 3 ERKAM-Clinical-Engineering Research and Application Center, Erciyes University, 38039, Kayseri, Turkey; 4 ERFARMA-Drug Development and Implementation Center, Erciyes University, 38039, Kayseri, Turkey; 5 Department of Pathology, Faculty of Veterinary and Animal Sciences, The Islamia University of Bahawalpur, Pakistan; 6 Department of Pharmaceutical Sciences, Superior University, Lahore, Pakistan; 7 Department of Pharmaceutics, Faculty of Pharmacy, The Islamia University of Bahawalpur, Pakistan; 8 Department of Microbiology, School of Basic Medical Science, Xinxiang Medical University, Xinxiang China; 9 Department of Pharmacology, University of Health Sciences, Khyaban-e-Jamia Punjab, Lahore, Pakistan; 10 Institute of Pharmaceutical Sciences, University of Veterinary and Animal Sciences, Lahore, Pakistan; 11 Xi'an Mental Health Centre, Xi'an China; 12 National Institiute for Biotechnology and Genetic Engineering College, Pakistan Institiute Engineering and Applied Sciences (NIBGE-C, PIEAS), Faisalabad 38000, Punjab, Pakistan; 13 Department of Zoology Wild-life and Fisheries, University of Agriculture, Faisalabad, Pakistan; 14 Department of Pharmaceutical Technology, Faculty of Pharmacy of the University of Coimbra, University of Coimbra, Coimbra, Portugal; 15 REQUIMTE/LAQV, Group of Pharmaceutical Technology, Faculty of Pharmacy of the University of Coimbra,University of Coimbra, Coimbra, Portugal

**Keywords:** Euthyroid thyroid cancer, DGGE, High-throughput sequencing, Characterization, Gut microbiota.

## Abstract

Thyroid cancer in humans has a fast-growing prevalence, with the most common lethal endocrine malignancy for unknown reasons**.** The current study was aimed to perform qualitative and quantitative investigation and characterization of the gut bacterial composition of euthyroid thyroid cancer patients. The fecal samples were collected from sixteen euthyroid thyroid cancer patients and ten from healthy subjects. The PCR-DGGE was conducted by targetting the V3 region of 16S rRNA gene, as well as real-time PCR for *Bacteroides vulgatus, E.coli Bifidobacterium, Clostridium leptum* and *Lactobacillus* were carried. High-throughput sequencing of V3+V4 region of 16S rRNA gene was performed on Hiseq 2500 platform on 20 (10 healthy & 10 diseased subjects) randomly selected fecal samples. The richness indices and comparative diversity analysis showed significant gut microbial modification in euthyroid thyroid cancer than control. At phylum level, there was significant enrichment of Firmicutes, Verrucomicrobia, while a significant decrease in Bacteroidetes was detected in the experimental group. At family statistics, significant high levels of Ruminococcaceae and Verrucomicrobiaceae, while the significant lower abundance of Bacteroidaceae, Prevotellaceae, Porphyromonadaceae, and Alcaligenaceae was after observed. It also found that the significantly raised level of *Escherichia-Shigella, Akkermansia* [Eubacterium]_coprostanoligenes*, Dorea, Subdoligranulum,* and *Ruminococcus_2 genera,* while significantly lowered genera of the patient group were *Prevotella_9*, *Bacteroides* and *Klebsiella*. The species-level gut microbial composition showed a significantly raised level of *Escherichia coli in* euthyroid thyroid cancer. Thus, this study reveals that euthyroid thyroid cancer patients have significant gut microbial dysbiosis. Moreover, Statistics (P<0.05) of each gut microbial taxa were significantly changed in euthyroid thyroid cancer patients. Therefore, the current study may propose new approaches to understanding thyroid cancer patients' disease pathways, mechanisms, and treatment.

## Introduction

Human gut microbiota is defined as a crucial factor in determining an individual's normal body functioning and health status. The body physiological mechanism depends on the configuration of gut microbiota which remains consistent over the period of time. It may be modulated by different factors like ageing, food, and sickness 1. The human gut microbiota constitutes approximately 100 trillion bacteria contributing to the immune function, metabolism, nutrition, absorption 2, and defence mechanism against pathogens 3. The modulation of gut microbial composition act as a predisposing factor for different disease condition like colitis, inflammatory bowel disease, Crohn's disease, metabolic disorders including diabetes mellitus, asthma, smoking and obesity 4-6.

Cancer is characterized by abnormal/uncontrolled growth of cells and is among the deadliest diseases 7-10, with an estimated 11.5 million deaths by 2030 worldwide 11. The etiological findings of thyroid cancer are commonly manifested with thyroid nodules exhibiting significant clinical signs in diagnosis. Recently, the number of patients with thyroid problems has been observed a sharp rise in worldwide, particularly in women 12. The recent epidemiological data suggested that thyroid dysfunction is the 5^th^ leading cancer diagnosed in women 13. If the same developments are continued, thyroid malignancy may perhaps develop into fourth leading carcinoma of the United States in 2030 15. The occurrence of thyroid malignancy in several European states also shows a similar tendency to the United States and followed in China 14. Thyroid cancer is commonly found in malignant endocrine carcinoma classified into 5 distinct types 15. The most prevailing thyroid cancer is papillary thyroid carcinoma (PTC), which is approximately about (80-85%) thyroid malignant patients in well-advanced nations 16. In PTC, the genetic modulations predispose the activation of 17 serum thyroid-stimulating hormone (TSH), thus generating malignant thyroid nodules. Many factors may cause many thyroid disorders, including estrogen, BMI ethnicity, abnormal iodine consumption and radioactivity 18-22. It has been described that the microbial composition of the gut could be altered by abnormal thyroid function 23. Numerous experimental works have been established that both Graves' disease and Hashimoto's thyroiditis are linked with gut microbiota 24, 25.

**Scheme 1:**
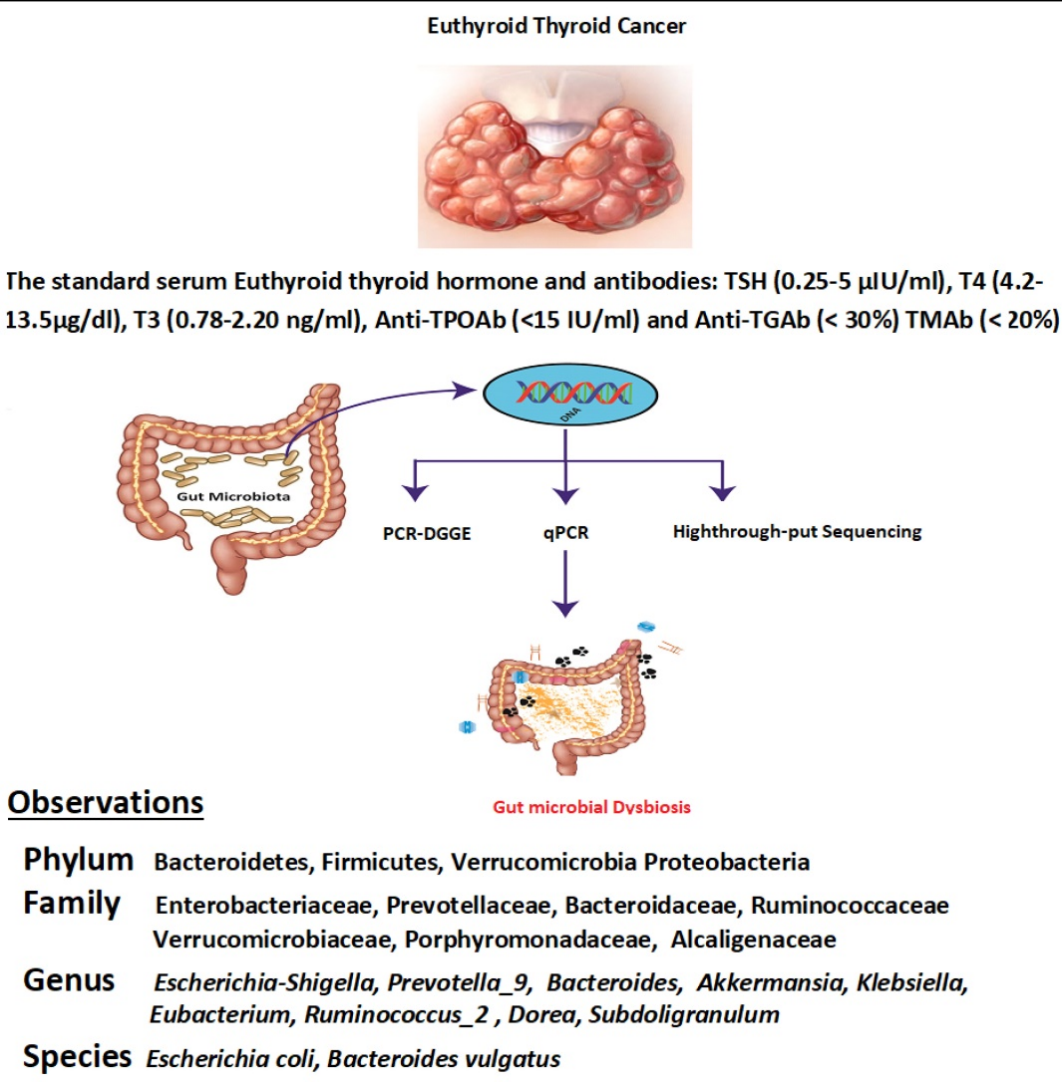
The whole study methodology and results clearly indicate gut bacterial dysbiosis in euthyroid thyroid cancer patients.

Furthermore, the traditional role of the thyroid gland in the immune system plays a vital role in the development of thyroid cancer. The recent experimental investigations have elaborated on the intestinal microbiota contribution to host immunity and body homeostasis 26. The current study was planned to determine the modification in diversity and similarity of intestinal microbiota qualitatively and quantitatively in euthyroid thyroid cancer patients with normal circulating antibodies compared to healthy subjects. Gut microbial diversity and similarity of euthyroid thyroid cancer patients were monitored by applying metagenomic High-throughput sequencing, PCR-DGGE, and qPCR. The findings revealed the significant difference in gut microbial composition with some distinctive gut bacteria portraying significantly elevated or lowered richness against healthy subjects. However, the association between gut microbiota and euthyroid thyroid cancer patients with normal level thyroid circulating antibodies has not been reported yet. The current study thus facilitates the explanation of the overall gut microbial composition of euthyroid thyroid cancer patients, as described in Scheme 1.

## Methodology

### Ethics statement

The volunteer wrote down a semi-structured detailed questionnaire as per the rules of Xi'an Jiaotong University (School of Medicine), the Ethics Committee.

### Sample collection

Fecal samples were collected in a sterilized cup from 16 thyroid cancer patients (8 males + 8 females) having euthyroid (aged linking 30 to 50 years) and 10 healthy volunteers (5 males + 5 female) (having the same age between 30 to 50 years). The patients with euthyroid thyroid cancer were diagnosed by set protocols of department of Endocrinology and Metabolic diseases, School of Medicine Xian Jiatong university 27. The normal standard antibodies and serum thyroid hormone levels are T3 (0.78-2.20 ng/ml), TSH (0.25-5 μIU/ml), T4 (4.2-13.5μg/dl), Anti-TGAb (< 30%) TMAb (< 20%), and Anti-TPOAb (<15 IU/ml). The information sheet for every volunteer was constructed according to their medical status, nutritional habits, lifestyle, and thyroid cancer disease. This inquiry performa also includes individual physical weight, age, and gender. The fecal samples were transported on ice almost four hours after defection. The samples were stored at -80 ⁰C freezer in the lab for further pocess like DNA extraction. In the last 60 days of sample collection, neither of the patients nor the healthy volunteers had taken any antibiotics, probiotics, and prebiotics. Our study subjects were also free of any GIT disease.

### DNA extraction from the fecal sample

DNA was extracted from all thawed fecal samples of diseased and control using the QIAGEN Stool kit (Germany) according to established protocol. The first step of bead-beating was conducted for ½ min with 5000 rpm. Concentration of DNA was evaluated using Nano Photometer TM (IMPLEN, Germany) 28.

### PCR Amplification for DGGE

The fecal extracted bacterial DNA was used for analyzing PCR-DGGE. The linkage primers for V3 region were used to emplify the 16S rRNA gene (Table 1). A total of 50 µl PCR mixture (thermocycler ABI2720 USA) was used to amplify the DNA sequence through touchdown PCR programming: Initial start was done with denaturation of PCR mixture at 95 ºC for 5 min that further include 10 extra cycles and followed by a final extension. The gene bands were assessed by using 1.5% agarose gel electrophoresis, and visualized under UV light after dipping in the Ethidium bromide solution 29.

### Denaturing gradient gel electrophoresis (DGGE)

The DGGE was performed by using universal mutational (Bio-Rad, USA) analysis. In brief, Amplified PCR microbial DNA was loaded in 8% acrylamide DGGE gels in a container with 1×TAE buffer solution (linear denaturant grade 30~65%) with the constant optimal temperature at 60 ⁰C. DGGE was run for 13 hours at 90 V. The syngene Genetool (4.3.14) software was used to determine each sample's fecal microbial diversity and intensity strength by analyzing the total number of bands in DGGE. The Dice similarity coefficient was also assessed by using a similarity index. The algorithm clusters and arithmetic means (UPGMA) were applied to develop the unweighted pair group dendrogram 30.

### DGGE statisticals analysis with band configuration

**Figure 1:**
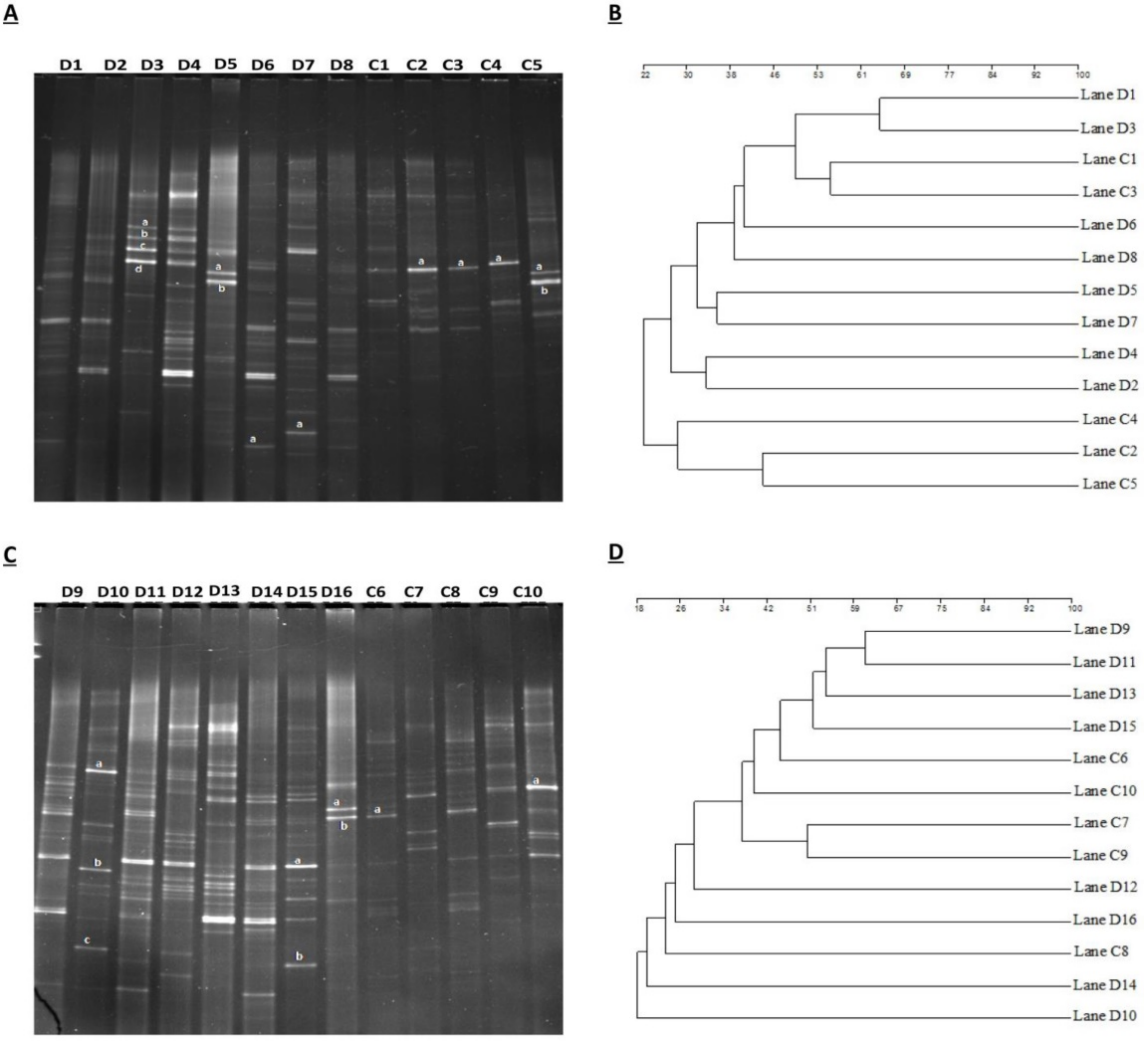
(A) Assembling the DGGE profile of euthyroid thyroid cancer patients (D1-D8) and control subjects (C1-C5). (B) UPGMA application for Cluster analysis between diseased (D1-D8) and controlled (C1-C5) groups. (C) DGGE depicts constructed between euthyroid thyroid cancer (D9 -D16) and control groups (C6-C10). (D) assembly analysis of euthyroid thyroid cancer(D9 -D16) and control (C6-C10) groups by applying UPGMA.“a” and “b” in figure (A) and (C) constitute the dominant bands of different patients. D or C represents the euthyroid thyroid cancer patients and control subjects, respectively.

Bands intensity of DGGE profiles and bands number were evaluated by computed Syngene software. Bacterial diversity in DGGE profiles was estimated by using *(H¹)* Shannon Weaver diversity index 31, 32. Similarity index and DGGE profile's cluster analysis were done through the UPGMA method (band-based Dice similarity coefficient) 33. GraphPad prism 7 and Microsoft Excel (2010) were used to analyse the Shannon-Weaver and similarity index, where (P<0.05) was considered as statistically significant. Also, the Similarities among DNA samples were indicated by the dendrogram, shown in Figures 1B and 1D.

Shannon Weaver diversity index (*H'*) was calculated by using the following formula.

Shannon-Weaver index (H¹) =
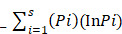


### Excision of bands and sequencing

From DGGE gel profile prominent bands were cut with the help of sterile scalpel blade. The excised polyacrylamide DNA gel bands were kept in a 2 ml tube. 50 μl of distelled water was added to the tube and placed at 37 ºC for 30 min. After centrifugation, 8 μl of DNA water was used as a template and similar primers (without GC-clamps) were applied for amplification of V3 region 16S rRNA gene 34. The sequencing of the amplified PCR-DNA was evaluated through ABI-3500xL. Obtained sequences were carefully analyzed by applying basic local alignment searching tool (BLAST) to identify the species or genus.

### Real-time PCR

The Bio-RAD CFX96 (USA) protocol kit was followed for real-time PCR. A total of 20 μl reaction mixture was used for Real-time PCR. The sample includes 2 μl of genomic DNA, 1 μl each of forward and reverse primer along with 10 μl of Sybr green adding in 6 μl of water, thus making the 20 μl of reaction mixture for loading. The details of linkage primers for Real-time PCR are shown in Table 1 35*.* The bacterial species* Bifidobacterium* (CICC.6186), *E.coli,* NWS* Lactobacillus* (taken from our lab), *Clostridium leptum* (YIT.6169),* Bacteroides vulgatus,* (CICC.22938) were used as standard bacterial strains. The average mean values were retrieved after performing the Real-time PCR thrice. The result was an estimation of the mean logarithm in the fecal sample of genomic PCR amplicon, where copy number present in 1g of the fecal mass.

### High-throughput sequencing and data analysis

The Ilumina based Hiseq protocol was followed for sequencing the paired-end. The data was retrieved and aligned by using QIIME and FLASH software. The QIIME (V1.7.0) software was used for diversity analysis like Simpson and Shannon diversity index and Good's coverage, ACE and chaol. The metagenomic high-throughput sequencing procedure was conducted based on the random reaction of 20 fecal samples. These include 10 samples collected from euthyroid thyroid cancer patients and the other 10 received from healthy subjects. The V3+V4 region of 16S rRNA gene was adjoined with linker primers: 806R (GGACTACHVGGGTWTCTAAT) 515F (GTGCCAGCMGCCGCGGTAA) primers for the construction of amplicon taxonomic libraries 36. The data was retrieved and aligned by using QIIME 37 and FLASH 38 software. The UCLUST procedure 37 was employed to aggregate the bacterial DNA sequences in operational taxonomic units (OTUs) at the level of 97% identity threshold. The taxonomic position of each OTU was allocated by using the RDP classifier 39. The QIIME software was used for diversity analysis like Simpson as well as Shannon diversity index along with chaol, Good's coverage and ACE. Moreover, the OTUs data tables retrieved through QIIME pipeline which were incorporated with MEGAN4 softwear and mapped with taxonomic database of NCBI 40. The gut microbial population composition is witnessing the significant alteration. UniFrac distances were calculated by applying QIIME. PCA (Principal component analysis) and NMDS (non-metric multi-dimensional scaling) estimation were used to observe the dissimilarities and similarities of variables which are represented in paired wise distances between the study and control groups. It showed through stat packages, ggplot2 package and WGCNA packages, in software R (Version 2.15.3). The differences of alpha diversity were compiled by using a nonparametric unpaired t-test by using Graphpad prism 7 (statistic software) and Microsoft Excel (2010).

## Results

### Statistics and DGGE profiles analysis in euthyroid thyroid cancer and healthy group

Analytical and experimental process of DGGE (denaturing gradient gel electrophoresis) was performed by using amplified PCR mixture employing universal primers of V3 region of 16S rRNA gene of euthyroid thyroid cancer and healthy control groups. In figure 1A, D1-D8 shows samples of euthyroid thyroid cancer patients and C1-C5 healthy controls. Similarly, in Figure 1C, the D9-D16 shows the samples of euthyroid thyroid cancer patients and C6-C10 healthy controls. Since the position, bands' strength intensity, and numbers were diverse among all fecal DNA samples that depicted the diverse intestinal microbial fingerprints. A total of 225 bands were identified by using the Syngene software 41, in 16 tracks of euthyroid thyroid cancer patients with an average band (14.1 ± 3.21). Moreover, a total of 86 DGGE bands were identified in 10 tracks of healthy subjects averaging (8.6 ± 2.55), which was significantly different (P< 0.001) between euthyroid thyroid cancer and healthy control groups. These results indicate that the increased band numbers demonstrate the increased diversity as well as bacterial overgrowth in euthyroid thyroid cancer patients group. To analyze, the diversity of stool microbiota in euthyroid thyroid cancer patients and healthy group, Shannon weaver (*H¹)* diversity index showed (3.225 ± 0.422 vs 2.542 ± 0.432) a significant (P< 0.003) in intestinal bacterial diversity alteration between euthyroid thyroid cancer and healthy subjects. *(H¹)* Shannon Weaver diversity index values were elevated in euthyroid thyroid cancer patients compared to healthy subjects, depicting significant gut bacterial overgrowth in euthyroid thyroid cancer patients. The similarity level of all the gut bacteria in DGGE gel profiles was assessed through the Dice similarity coefficient (UPGMA) dendrogram explained in Figures (1B and 1D). The band intensity-based numeral values of the Dice similarity coefficient between euthyroid thyroid cancer and healthy groups, along with mean similarity index (0.319 ± 0.141) and (0.288 ± 0.130) respectively, are shown in Table 2. When all the statistical values of each sample of euthyroid thyroid cancer and healthy control were calculated and analyzed through mean similarity index and Dice similarity coefficient, they were (0.269 ± 0.125). These findings illustrated that it was exhibited lesser in intergroup compared to intragroup, thus presenting dissimilarity of gut microbial composition in euthyroid thyroid cancer patients in contrast to the control subjects.

### Dominant bands sequencing results analysis

The totals of 22 gel bands were excised by two DGGE gel profiles. In Figure 1A, 13 gel bands were cut from the DGGE profile for quantitative analysis. The resolution capability of DGGE gel profile bands was confirmed in different tracks, but in a similar position, the gel bands D5a and C5a were sequenced after excision that was identified as *Prevotella copri* with 96 % similarity. Furthermore, from Figure 1C, 9 bands were cut and assessed the resolution capability of DGGE gel composition; bands D16b and C6a were sequenced and detected as *Bacteroides vulgatus* having 98% similarity. The taxonomic identity of other bands of the DGGE profile has depicted in Table 3. Sequencing results were evaluated and analyzed by deploying BLAST software, and findings have confirmed the prevalence of phylum Firmicutes, Proteobacteria, and Bacteroidetes as prominent presence. Sequencing results of excision band from two DGGE gel profiles, also depicted in Table 3, opportunistic bacteria are prevalent (Escherichia coli, Proteus mirabilis, Pseudomonas cremoricolorata, Prevotella oulorum, Faecalibacterium prausnitzii, Phascolarctobacterium sp, Alistipes putredinis, Shigella dysenteriae, Bacteroides pyogenes, Bacillus sp. Klebsiella sp, Enterobacter sacchari, Parabacteroides distasonis) in euthyroid thyroid cancer patients.

### Real-time PCR Amplification

The Bacteroides vulgates, Bifidobacterium, Lactobacillus Clostridium leptum*,* were enumerated by Real-time PCR. The copy numbers of Bifidobacterium (5.75 ± 0.87 vs. 6.73±0.87) were lessened significantly (P < 0.005), also copy numbers of Lactobacillus (6.19 ± 0.98 vs 6.98 ± 0.99) were lowered significantly (P < 0.029) in the disease group. Conversely, Bacteroides vulgatus (5.77 ± 0.86 vs 6.59 ± 0.82) were found significantly (P <0.011) reduced in euthyroid thyroid cancer patients. Copy numbers of Escherichia coli (5.60 ± 0.78 vs. 4.89 ± 0.74) were significantly (P < 0.016) increased in patients. Moreover, the copy numbers of Clostridium leptum (4.05 ± 1.07 vs 3.75 ± 1.11) (P < 0.249) had a non-significant rise in the fecal samples of euthyroid thyroid cancer patients in contrast with healthy subjects. The results are shown in Table 4.

### High-throughput sequencing of gene analysis

**Figure 2:**
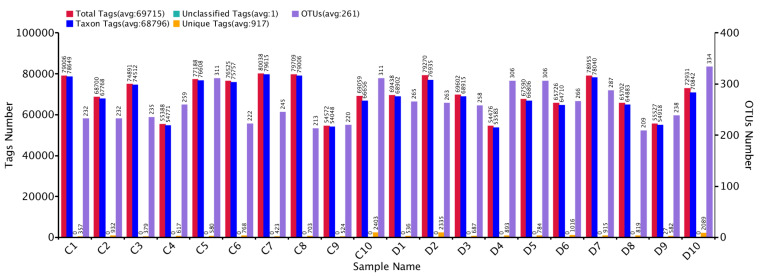
Euthyroid thyroid cancer observation of Tag number and OTUs analysis with comparison of control, Tag number and OTUs were estimated at the level of (97 %) similarity

Comparative sequencing amplicons of PCR were computed with 1,731,168 at the position of V3+V4 of the 16SrRNA gene, from 10 euthyroid thyroid cancer and also10 from healthy controls. High-throughput sequencing reads 1, 438,586 (control 736,933 and disease 701,653) were passed, having an average per sample (72,169) for quality assurance and analysis. The taxon tag was estimated (Ave. 68796.2) in both euthyroid thyroid cancer and healthy control groups. Total unique tag counted in the study and healthy groups were 10, 656 and 7,686, respectively (Ave. 917.1 in entire samples). Operational texsonomic unit (OTU) numbers were assigned which is 52,12 (healthy 24,80 and patients 2,732) average/sample (260.6) in current study. The high-throughput unique tag was 18,342 from study and healthy groups, demonstrating the entire phylotypes of current experimental work. The OTU clustering and annotation results of each sample are comprehensively calculated. The results are shown in Figure 2. The average length of the sequence was estimated 418 bp after removal of linkage primers.

### Intestinal bacterial diversity analysis

**Figure 3:**
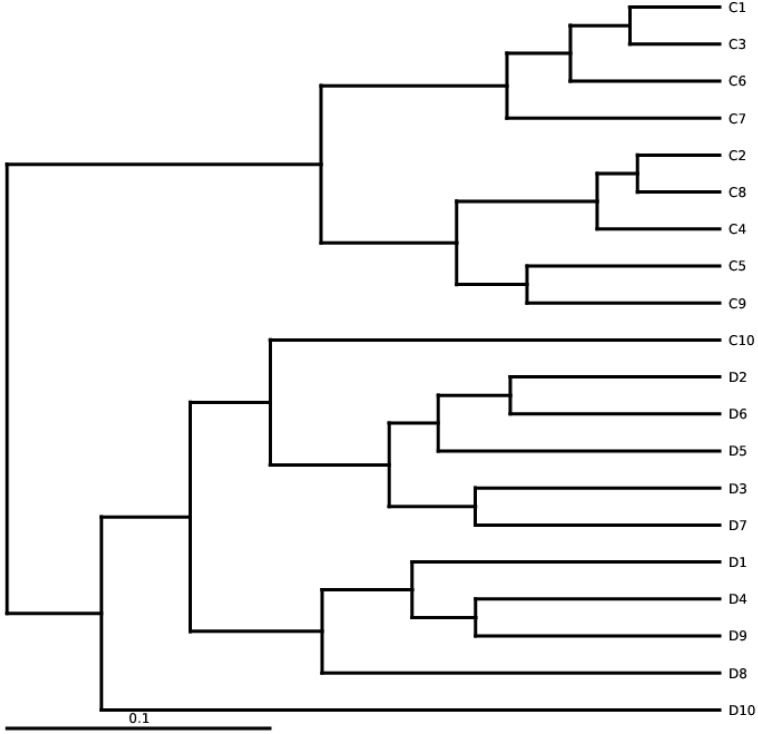
Diversification among euthyroid thyroid cancer samples of High-throughput sequencing. UPGMA is based on weighted UniFrac distances. D and C denotes the euthyroid thyroid cancer patients and controlled group, respectively.

Bacterial community diversity and richness were estimated at a similarity level of 97%. Alpha diversity, as computed by Simpson and Shannon diversity, PD Tree, algorithm ACE, observed species, and Chao1, were found significantly higher in euthyroid thyroid cancer with a comparision of healthy volunteers. The level of bacterial diversity assessment in study and control is described in Table 5. Additionally, the analysis of alpha diversity exhibits an elevated level in euthyroid thyroid cancer patients compared to controls. The elevated diversity depicted a strong intestinal bacterial overgrowth in the patients' group compared to healthy volunteers. Samples of intestinal bacterial DNA in each group were distributed in two distinct clusters, constructed on weighted UniFracs distance shown in Figure 3**,** which is also analogous to the arrangement of PCR-DGGE of euthyroid thyroid cancer and normal volunteers.

**Figures 4:**
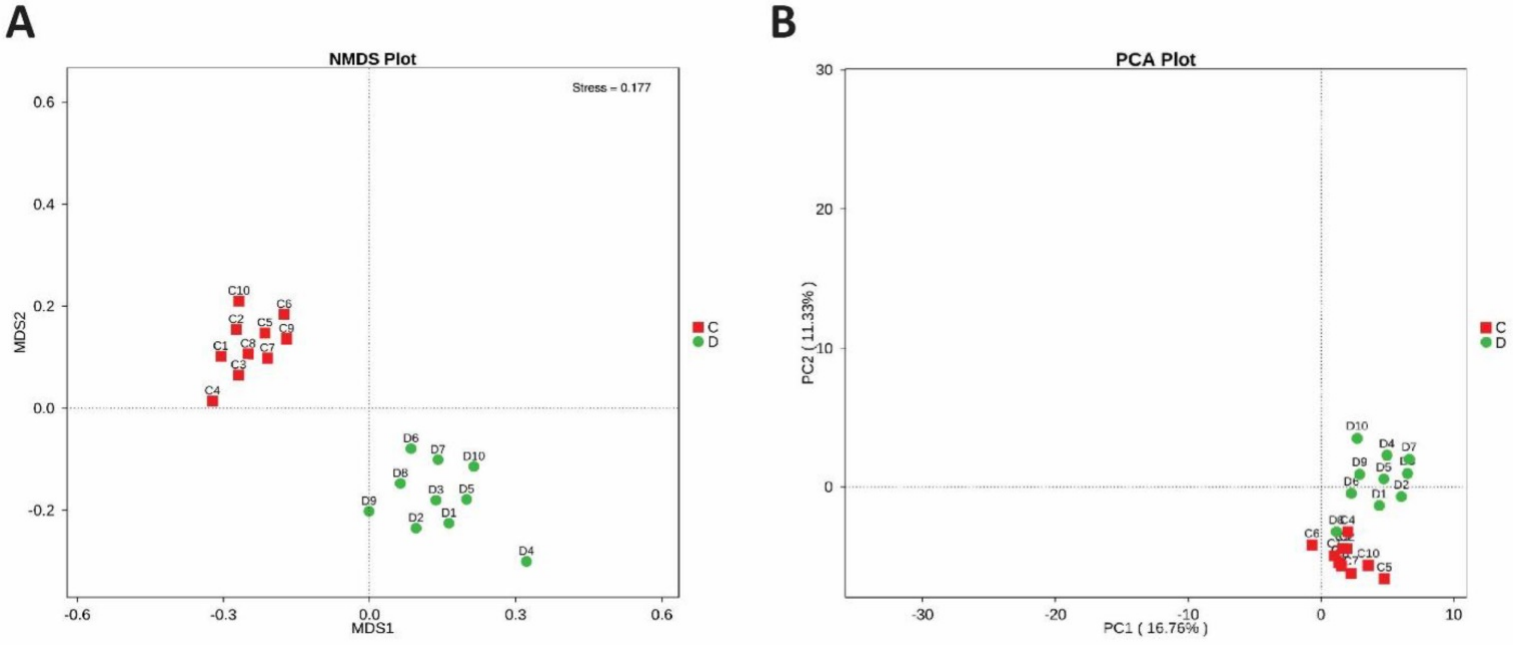
** (A)** Beta diversity between diseased and healthy subjects. PCA plots which are obtained from Highthrough-put sequencing of fecal microbial DNA samples. (**B)** NMDS plot between study and control bacterial DNA samples. Each dot in the plot indicates an individual fecal bacterial DNA Samples of patient and control group

To determine the microbial diversity between the healthy and study groups, the beta diversity was estimated. Non-metric dimensional scaling (NMDS) and OTU number based Principal-component analysis (PCA) were performed, which clearly illustrate the alteration of the intestinal bacterial composition of two groups, shown in Figures 4A and 4B.

### Phyla level intestinal bacteria

Intestinal bacterial taxa had a percentage over 0.5% - 1%, considered in the present study, at the phylum, family, genus and species level.

**Figure 5:**
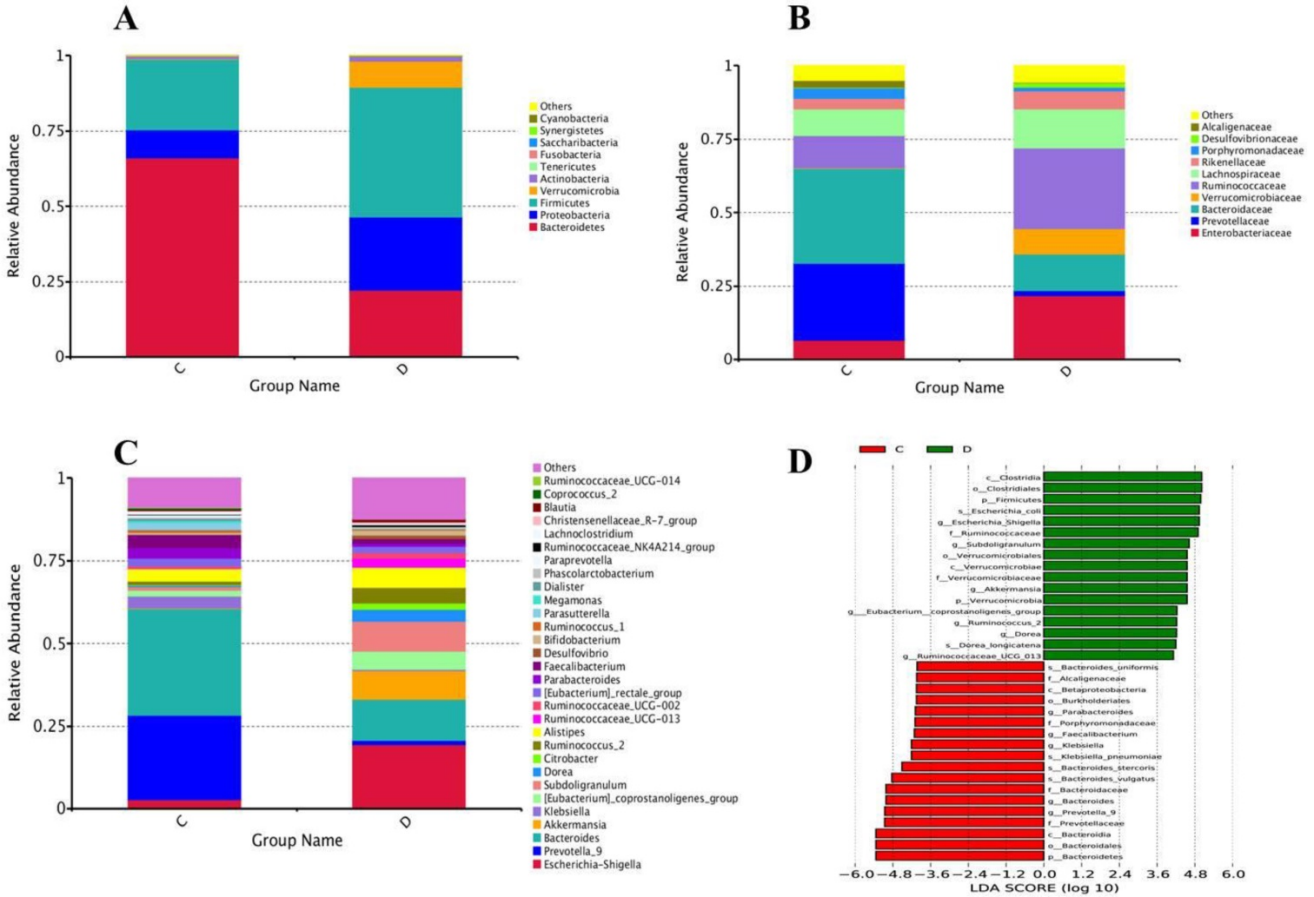
** (A)** Configuration of Gut microbiota at phyla levels from High-throughput sequencing results. The excessive occurrence of the most prevalent phyla in euthyroid thyroid cancer and control D and C designated as euthyroid thyroid cancer patients and control group, respectively, **(B)** High-throughput sequencing findings of gut bacterial conformation at family level. The relative plentiful of the most profoundly found families in euthyroid thyroid cancer and healthy controls. D and C represent euthyroid thyroid cancer and control group, respectively,** (C)** The genera levels gut bacterial compositions from High-throughput sequencing results. The relative abundance of the most prevalent genera in euthyroid thyroid cancer and healthy control. D and C represent euthyroid thyroid cancer and control group, respectively,** (D)** LDA (linear discriminant analysis) value distribution histogram was applied to find the most altered gut bacterial taxa abundance between euthyroid thyroid cancer patients D and control subjects C.

At the level of phylum, a total of 15 phyla were found in sequencing; among the 10 topmost phyla, significantly increased phyla abundance in the diseased group were Firmicutes and Verrucomicrobia while non- significantly raised in Proteobacteria and Actinobacteria. However, Phylum Bacteroidetes in the experimental group was significantly reduced compared to healthy volunteers, depicted in Figure 5A. Statistics of the 10 most prevalent phyla in Table 6A illuminated significant quantitative difference between the two groups.

### Intestinal bacterial composition at a family level

Family level sequencing, 75 diverse families were sequenced through Illumina based sequencing, in 10 topmost families, taxa richness of Ruminococcaceae, and Verrucomicrobiaceae were significantly elevated while non-significantly increased in families Enterobacteriaceae, Lachnospiraceae and Rikenellaceae in the study group with the comparison of control, depicted in Figure 5B. Among all these families, the community abundance of Bacteroidaceae and Prevotellacea was significantly reduced in the study group compared to healthy volunteers. Percentage data statistics in euthyroid thyroid cancer group displayed a significant quantitative variation of families shown in Table 6A.

### Genus level intestinal bacterial distribution

Genera level highthrough-put sequencing characterized the abundance of 211 diverse genera. Among 30 topmost sequenced genera, there was significantly raised genera prevalence of Escherichia-Shigella, [Eubacterium]_coprostanoligenes, Subdoligranulum, and Ruminococcus_2 in the study group with the comparison of healthy group depicted in Figure 5C. Conversely, significantly lowered genera in the patients' group were Bacteroides, Klebsiella and Prevotella_9*.* Genera abundance statistics in two groups were gathered in Table 6B. Euthyroid thyroid cancer has a specific effect on intestinal bacteria, in particular, Phylum Firmicutes Verrucomicrobia, Proteobacteria, Bacteroidetes and Actinobacteria, families Ruminococcaceae, Verrucomicrobiaceae, Prevotellacea and Bacteroidaceae genera Escherichia-Shigella, [Eubacterium]_coprostanoligenes, Subdoligranulum Ruminococcus_2, Prevotella_9, *Bacteroides* and Klebsiella*.* The disease also dramatically influences the intestinal bacteria, which may change the health status of an individual due to the alteration of intestinal bacterial composition.

### Gut bacterial composition at the species level

Species-level intestinal bacterial community patterns are demonstrated in Table 7. The species finding illustrate the significant differences between euthyroid thyroid cancer and healthy volunteers. However, the levels of Escherichia coli in euthyroid thyroid cancer patients have been raised significantly when compared to healthy volunteers.

The linear discriminant analysis (LDA) value distribution histogram demonstrates the taxa differences between the two groups. Taxa with significant differences in abundance in euthyroid thyroid cancer and control groups, shown in Figure 5D, and the length of the histogram represents the size and impact of the different Taxa.

The final data obtained from the results analysis of metagenomic DGGE and High-throughput sequencing confirms the similar bacterial taxa prevalence. However, High-throughput sequencing is a highly sensitive, authentic, and much reliable technique than DGGE to study the intestinal bacterial taxa. In conclusion, the results agree and affiliate the intestinal bacterial data generated by three molecular procedures.

## Discussion

Thyroid cancer is an endocrine system malignancy which is progressively rising in the last few decade that is to be believed due to better diagnostic facilities.^42^. Recent studies have been illustrated that gut microbiome has great importance and role in driving the different types of malignancies, including lung, breast, intestine and esophageal cancer 43, 44. The human gut microbiota role is also critical in protecting the body's defense mechanism by employing trophic activity 45. The gut microbiota and its metabolites like short-chain fatty acids have great impact in normal functioning of thyroid gland. It shows an existence of gut-thyroid axis and gut bacterial dysbiosis in autoimmune thyroid diseases such as Hashiomot's thyroiditis and Graves' disease 24, 25, 46.

The intestinal bacterial confirmation has been underlined in various disease situations, i.e., melanoma and diabetes too 4. The DGGE gel profile findings were elucidated by illuminating the sequencing of prominent bands, High-throughput sequencing, and Real-time PCR. The statistical data in α diversity, nonparametric Simpson, Shannon, Chao1, observed species, and ACE algorithm were found significantly elevated in the patients' group compared to the control group 47. Likewise, the diversity of gut bacterial population assessment in DGGE banding profiles and High-throughput sequencing analysis were found elevated in euthyroid thyroid cancer patients. Therefore, this increased level of gut microbiota evident the notable overgrowth in patients compared to the control group. However, these raised findings and interpersonal variations parallel to microbial results of skin, vagina, and gastrointestinal tract 48, 49.

The statistical data interpretation of intestinal microbial similarity index of euthyroid thyroid carcinoma group in DGGE banding profile configuration of intra-groups was measured to be significantly elevated; this indicated the bacterial overgrowth in the gut of study group. The comparative data analysis of diversity and similarity index found lesser in intergroup with the comparison of intra-group that are aligned with preceding research literature 50, indicating the variation in the composition of intestinal microbiota in euthyroid thyroid cancer patients with comparison of normal control. Hence, the diversity as mentioned above, findings elucidate a significant dissimilarity in the composition of intestinal bacteria between patients and healthy control groups. The statistical data showed significant quantitative and qualitative alteration between study and healthy groups.

At phylum level study, Firmicutes exhibited a significantly higher trend while low in Bacteroidetes in euthyroid thyroid cancer patients with comparison of control that is compatible with reported research in gut microbial alteration, endocannabinoid tone system and chronic pain with vitamin D mediated deficiency 51. The reported meta-analysis of intestinal bacteria related to IBD and obesity showed that the percentage of Firmicutes to Bacteroidetes is not a constant feature that is distinct between lean and obese intestinal bacteria 52. The current study illustrates a higher level of Proteobacteria, which is in accordance with prior work of proteobacteria as risk-factor in abdominal pain patients of the post-cholecystectomy syndrome 53. It has also been reported that Proteobacteria has a crucial role in inflammatory bowel disease, metabolic disorders, asthma, and obstructive pulmonary diseases 54. Our work indicates the significant increased abundance of phylum Verrucomicrobia which agrees with the reported literature of abnormalities of blood pressure associated and guts microbiota of children with anomalies of the urinary tract and kidney 55. Furthermore, Verrucomicrobia is related to blood pressure abnormalities in the initial stage of CKD children 55.

At the family level, our study showed an increased level of Enterobacteriaceae, agreeing with the reported work of altered gut microbiota in vitamin D deficiency-mediated chronic pain 56. It has been documented that pathogens of family Enterobacteriaceae involve in nosocomial pneumonia which is approximately1/3 of reported cases 57. Our work showed a significant decrease of family Bacteroidaceae which is aligned with published literature of intestinal microecology in primary Sjogren's Syndrome patients 58. The current study showed a significantly higher level abundance of family Ruminococcaceae, while significantly decreased family Prevotellaceae, which is parallel with reported work of diabetes type 2 and changes in intestinal bacteria with supplementation of diet 59.

Family Verrucomicrobiaceae is significantly enriched in study group, which is aligned with previous work of variation of the intestinal microbiota of the Chinese population with Parkinson's disease 60. Our study depicted a significant abundance of Eubacterium and Akkermansia, which aligns with the existing work of altered intestinal microbiota in chronic pain endocannabinoid tone systems with vitamin D deficiency 56. However, In advanced periodontal disease*,* Eubacterium may contribute to making about half of the microbiota and express a significant relationship with the disease 61. It has been published in numerous studies of humans and mice that the increased abundances of Akkermansia are linked with patients of post-RYGB 62.

Moreover, genera Prevotella_9 were significantly diminished in the study group. Decreased level of Prevotella has been publicized in types1 diabetes and autism with intestinal bacteria 63, 64, while Escherichia-Shigella genera were increased in the study group, which agrees with reported preliminary research of gut bacterial relationship with autism problems 65. Current study results depicted the lowered level of the Prevotella genus; however, the existing research literature shows the Prevotella dominance in intestinal microbial composition, which has a positive effect on the host's metabolism 66. Prevalence of the Prevotella genus is considered a useful gut bacteria which help in digestion of plant-based food material. Also, the gut microbiota has been associated with numerous inflammatory conditions and diseases 67, 68. Our results exhibited a significant raised level of Escherichia-Shigella in euthyridthyroid cancer patients with a comparison of healthy control*.* Previously, it was documented that Escherichia-Shigella can produce Shiga-toxin, which may cause thrombocytopenia septicaemia, hemorrhagic colitis, gastrointestinal inflammations, particularly in the ileocolonic region, hemolytic uremic syndrome (HUS), problems in urinary duct track 69. The current study indicated a significantly higher prevalence of genus Escherichia-Shigella*,* particularly *Escherichia coli* species, which could be the strongest contributing agent in intestinal bacteria of euthyroid thyroid cancer patients. Besides, *Escherichia coli* (ubiquitous) is responsible for triggering predominant infections, i.e. urinary tract infections (UTIs) and foodborne illnesses 70.

Current research work described the significan alteration in the abundance of the phylum to genus and species-level in experimental samples, which demonstrated the clear disparity between euthyroid thyroid cancer patients and control groups. Furthermore, the bacterial community and species-level comparison also unveiled a significant variation of gut bacterial composition in study group as compared of healthy control 71. These research findings further elaborate that euthyroid thyroid cancer plays a critical role in altering intestinal physiology that may cause the modification in the composition of gut microbiota. Likewise, such variations in intestinal microbial composition may trigger the disease complications 72.

The clinical signs of thyroid cancer manifested with thyroid nodules. However, Serum circulating antibodies, i.e., anti-thyroglobulin antibodies, anti-thyroid peroxidase and thyroid hormones in euthyroid thyroid cancer patients and healthy volunteers, are shown in (Table S1 and Table S2). The normal serum results of thyroid cancer patients in Table S1 showed euthyroid in thyroid cancer patients. So, it may be hypothesized that euthyroid thyroid cancer might alter the intestinal bacterial composition, in particular, Phylum Bacteroidetes, Firmicutes, Verrucomicrobia, family Enterobacteriaceae, Prevotellaceae, Bacteroidaceae, Verrucomicrobiaceae Ruminococcaceae, genera Escherichia-Shigella, Prevotella_9, Bacteroides, Akkermansia, Klebsiella, Eubacterium and Escherichia coli species, also largely disturb the intestinal bacteria. The current study on bacterial intestinal alterations between the euthyroid thyroid cancer group and healthy counterparts was very interesting because there is no direct connection and the straight relationship between euthyroid thyroid cancer and gut bacteria. Thus, the current study results further intricate the diverse intestinal bacterial composition between euthyroid thyroid cancer and normal healthy counterparts. These bacterial alterations may disturb the host's health status. Nevertheless, disease progress has no direct linkage with the gastrointestinal tract 73.

The Real-time PCR experiment was performed to investigate the quantitative gut microbial changes 74. Statistical data showed a significant reduction of Lactobacillus in the study group, hence parallel with previously reported research 75. The food supplement probiotics belong to genera Lactobacillus and incorporate physiological health benefits in the body 76. Moreover, Lactobacillus has been observed a reduced trend in diseases like colorectal cancer 77. In the human gut, Lactobacillus has great importance in the maintenance of selenium levels inside the cell. Selenium plays a crucial role in producing thyroid hormone and avoiding the oxidative destruction of the thyroid gland 78, 79. Many studies also exhibited good effects against anti-atherogenic, anti-obesity and anti-inflammatory body response 80. Different strains of lactobacillus have excellent antimicrobial characteristics in the human body to protect against uropathogens 81.

There was a significant decreased level of Bacteroides vulgates in the study group, which is steady with the reported literature of viral diarrhea with intestinal bacteria, while a significant increased level of Escherichia coli 82, 83. Numerically Bacteroides vulgatus species is the predominant Bacteroides of human intestinal bacteria, which comprise a beneficial but complex association with its host and the avoidance of gut colonization 84, 85. Data generated from PCR-DGGE and Illumina-based High-throughput sequencing analysis is suitable for characterizing intestinal bacteria. However, PCR-DGGE has been observed as a semi-quantitative technique; banding profile assessment which may not accurately illustrate the targeting taxa abundance of intestinal bacterial community 32. Moreover, Illumina-based High-throughput sequencing is a highly sensitive, most advanced, and pretty much reliable technique to study and investigate the intestinal bacterial ecology 73. Furthermore, the technique like PCR-DGGE might be applied as a routine basic laboratory technique to detect the significant modulation of gut bacterial taxa because it is lesst time-taking and low-cost experimental method.

## Conclusion

A difference in composition of gut microbiota was found between euthyroid thyroid cancer patients and healthy counterparts. More precisely, there is a significant alteration in intestinal bacterial taxa abundance of the study group compared to healthy groups. The bacterial estimation analysis of taxa diversity exhibits a higher level of presence of intestinal bacteria in the euthyroid thyroid cancer group than controls, which shows the bacterial dysbiosis and overgrowth in the euthyroid thyroid cancer patients group. Consequently, the additional multicentre study approach has been needed to apprehend the basic underlying process and mechanism of bacterial dysbiosis in the intestine of euthyroid thyroid cancer patients.

## Supplementary Material

Supplementary tables.

## Figures and Tables

**Table 1:** Primers used in Real-time PCR and PCR-DGGE

Target bacteria		Primer Sequence (5¹-3¹)
PCR-DGGE Primer		
341-F		CCTACGGGAGGCAGCAG
534-R		ATT ACCGCGGCTGCTGG
341FG		CGCCCGCCGCGCGCGGCGGCGCGGGGCGGGGGCACGGGGGGCCTACGGGAGGCAGCAG

Real-Time PCR Primer		
*Bifidobacterium* (550 bp)	Bifid F	CTC CTGGAAACGGGTGG
	Bifi-R	GGTGTTCTTCCCGATATCTACA
*Lactobacillus* (250 bp)	Lact F	CTCAAAACTAAACAAAGTTTC
	Lact R	CTCAAAACT AAACAAAGTTTC
*Bacteroides vulgatus* (287bp)	BV- F	GCATCATGAGTCCGCATGTTC
	BV-R	TCCATACCCGACTTTATTCCTT
Escherichia coli (287bp)	*E.coli*-F	CATTGACGTTACCGCAGAAGAAGC
	*E.coli*-R	CTCTACGAGACTCAAGCTTGC
*Clostridium leptum* (239bp)	C.lep-F	GCACAAGCAGTGGAGT
	C.lep-R	CTTCCTCCGTTTTGTCAA

**Table 2:** Gut microbial similarity and diversity of euthyroid thyroid cancer patients and healthy group

Groups	Diversity		Similarity	
	The number of Bands A	Shannon Index B	Intra-similarity C	Inter-similarity D
Disease group	14.1 ± 3.21	3.225 ± 0.422	0.319 ± 0.141	0.269 ± 0.125
Control group	8.6 ± 2.55	2.542 ± 0.432	0.288 ± 0.130	
P. Value	0.001	0.003	/	/

Significantly different results (unpaired t-test), with P<0.05 a. DGGE bands number produced by each sample. b. Shannon diversity index *(H¹*) was calculated with the help of all DGGE bands ( relative intensities) in each sample. c. Comparing DGGE band profiles with Dice similarity coefficients within the individual of a given group. d. Comparing DGGE band profiles with Dice similarity coefficients between members of euthyroid thyroid cancer and the healthy group.

**Table 3:** Sequencing of re-amplified PCR Amplicons excised bands from DGGE gel and identities were checked by BLAST database.

Selected Excised bands	Bacteria with the highest % homology	Sequence Accession number	Bacterial phyla	Gene bank number
D3a	Escherichia coli (93).	IAI39.	Proteobacteria	NZ_JH114216.1
D3b	Proteus mirabilis (94).	HI4320.	Proteobacteria	NC_010554.1
D3c	Pseudomonas cremoricolorata (98).	ND07.	Proteobacteria	NZ_CP009455.1
D3d	Prevotella oulorum (93).	F0390.	Bacteroidetes	NZ_JH114216.1
D5a	Prevotella copri( 96).	DSM 18205.	Bacteroidetes	NZ_GG703862.1
D5b	Faecalibacterium prausnitzii (96).	TDY5834930.	Firmicutes	NZ_CZBH01000014.1
D6a	Phascolarctobacterium sp (94).	YIT 12067.	Firmicutes	NZ_GL830850.1
D7a	Alistipes putredinis (99).	DSM 17216.	Bacteroidetes	NZ_DS499580.1
C2a	Bacteroides oleiciplenus (92).	YIT 12058.	Bacteroidetes	NZ_JH992946.1
C3a	Bacteroides uniformis (90).	CL03T00C23.	Bacteroidetes	NZ_JH724260.1
C4a	Barnesiella intestinihominis (98).	YIT 11860.	Bacteroidetes	NZ_JH815205.1
C5a	Prevotella copri (96).	DSM 18205.	Bacteroidetes	NZ_GG703862.1
C5b	Bacteroides stercoris (90).	ATCC 43183.	Bacteroidetes	NZ_DS499675
D10a	Shigella dysenteriae (98).	Sd197.	Proteobacteria	NC_007606.1
D10b	Bacteroides pyogenes (88).	JCM 10003.	Bacteroidetes	NZ_BAIU01000058.1
D10c	Bacillus sp. (94).	FJAT-25496.	Firmicutes	NZ_LMBY01000086.1
D15a	Klebsiella sp.(94).	NODE14.	Proteobacteria	NZ_LGIT01000014.1
D15b	Enterobacter sacchari (94).	SP1.	Proteobacteria	NZ_CP007215.2
D16a	Parabacteroides distasonis (97).	ATCC 8503	Bacteroidetes	NZ_JH815205.1
D16b	Bacteroides vulgatus ( 98).	ATCC 8482.	Bacteroidetes	NC_009614.1
C6a	Bacteroides vulgatus ( 98).	ATCC 8482.	Bacteroidetes	NC_009614.1
C10a	Bacteroides paurosaccharolyticus (91).	JCM 15092.	Bacteroidetes	NZ_BAJR01000054.1

**Table 4:** Real-time PCR quantification results in different gut bacteria

Bacteria	Healthy Subjects	Patients	P value
Bifidobacterium (10^4^)	6.73±0.87	6.30±0.90	0.123
Bacteroides vulgatus(10^9^)	6.59±0.82	5.77±0.86	0.011
Lactobacillus (10^5^)	6.98±0.99	6.19±0.98	0.029
Clostridium leptum (10^7^)	3.75±1.11	4.05±1.07	0.249
Escherichia coli (10^6^)	4.89±0.74	5.60±0.78	0.016

Results were presented as the average estimate of logarithms of fecal PCR target genetic amplicon copy numbers present in 1 g of feces, where (P < 0.05).

**Table 5:** Gut bacterial richness and diversity index, based on 97% similarity through High-throughput analysis.

Group	Observed Species	OTUs	Shannon	Simpson	Chao1	ACE	PD Tree	Evenness
Patients	255	248	4.62	0.876	273.50	279.39	22.02	0.357
Control	226.5	273.2	3.85	0.770	240.24	244.54	19.44	0.296
P. Value	0.042	0.067	0.011	0.0321	0.036	0.030	0.037	0.014

**Table 6A:** Gut microbial taxa at phyla and families level from High-throughput results

Taxa	Mean D	Mean C	p.value	q value	%D	% C
Phylum						
Bacteroidetes	0.2224	0.6605	0.0010	0.0040	22.24	66.05
Proteobacteria	0.2420	0.0941	0.1039	0.1662	24.20	9.41
Firmicutes	0.4305	0.2345	0.0050	0.0160	43.05	23.45
Verrucomicrobia	0.0862	0.0009	0.0010	0.0040	8.62	0.09
Actinobacteria	0.0167	0.0092	0.2947	0.3929	1.67	0.92
Tenericutes	0.0015	0.0000	0.0060	0.0160	0.15	0.00
Fusobacteria	0.0000	0.0008	0.0290	0.0515	0.00	0.08
Saccharibacteria	0.0002	0.0000	0.0070	0.0160	0.02	0.00
Synergistetes	0.0001	0.0000	0.0010	0.0040	0.01	0.00
Cyanobacteria	0.0002	0.0000	0.0250	0.0500	0.02	0.00
Others					0.01	0.00
Family						
Enterobacteriaceae	0.2169	0.0650	0.0679	0.2323	21.69	6.50
Prevotellaceae	0.0167	0.2624	0.0030	0.0325	1.67	26.24
Bacteroidaceae	0.1260	0.3238	0.0150	0.0949	12.60	32.38
Verrucomicrobiaceae	0.0862	0.0009	0.0010	0.0127	8.62	0.09
Ruminococcaceae	0.2750	0.1113	0.0050	0.0475	27.50	11.13
Lachnospiraceae	0.1323	0.0889	0.1259	0.3417	13.23	8.89
Rikenellaceae	0.0621	0.0378	0.2478	0.5717	6.21	3.78
Porphyromonadaceae	0.0125	0.0351	0.0070	0.0591	1.25	3.51
Desulfovibrionaceae	0.0144	0.0011	0.0010	0.0127	1.44	0.11
Alcaligenaceae	0.0020	0.0237	0.0010	0.0127	0.20	2.37
Others					5.58	5.02

Euthyroid thyroid cancer D and Control C, P<0.05

**Table 6B:** Gut microbial phylotypes at genus level from High-throughput results

Taxa	Mean D	Mean C	p.value	q value	% D	% C
Genus						
Escherichia-Shigella	0.1941	0.0265	0.0140	0.0824	19.41	2.65
Prevotella_9	0.0125	0.2568	0.0020	0.0193	1.25	25.68
Bacteroides	0.1260	0.3238	0.0160	0.0869	12.60	32.38
Akkermansia	0.0862	0.0009	0.0010	0.0125	8.62	0.09
Klebsiella	0.0031	0.0351	0.0340	0.1412	0.31	3.51
[Eubacterium]_coprostanoligenes	0.0564	0.0178	0.0290	0.1253	5.64	1.78
Subdoligranulum	0.0888	0.0105	0.0010	0.0125	8.88	1.05
Dorea	0.0377	0.0038	0.0010	0.0125	3.77	0.38
Citrobacter	0.0179	0.0020	0.4525	0.7260	1.79	0.20
Ruminococcus_2	0.0459	0.0109	0.0180	0.0953	4.59	1.09
Alistipes	0.0619	0.0377	0.2388	0.4907	6.19	3.77
Ruminococcaceae_UCG-013	0.0280	0.0024	0.0020	0.0193	2.8016	0.2437
Ruminococcaceae_UCG-002	0.0168	0.0060	0.1948	0.4302	1.6804	0.5981
[Eubacterium]_rectale_group	0.0181	0.0248	0.5335	0.7440	1.8073	2.4769
Parabacteroides	0.0092	0.0314	0.0080	0.0529	0.9193	3.1430
Faecalibacterium	0.0137	0.0385	0.0030	0.0254	1.3730	3.8462
Desulfovibrio	0.0112	0.0004	0.0010	0.0125	1.1250	0.0424
Bifidobacterium	0.0074	0.0143	0.2358	0.4900	0.7390	1.4255
Ruminococcus_1	0.0041	0.0082	0.5654	0.7684	0.4091	0.8187
Parasutterella	0.0018	0.0203	0.0240	0.1105	0.1752	2.0260
Megamonas	0.0003	0.0079	0.0010	0.0125	0.0338	0.7905
Dialister	0.0006	0.0061	0.0999	0.2750	0.0586	0.6062
Phascolarctobacterium	0.0021	0.0044	0.5604	0.7665	0.2124	0.4408
Paraprevotella	0.0026	0.0053	0.3516	0.6318	0.2646	0.5263
Ruminococcaceae_NK4A214_group	0.0051	0.0020	0.1369	0.3297	0.5058	0.1965
Lachnoclostridium	0.0052	0.0098	0.0420	0.1435	0.5250	0.9817
Christensenellaceae_R-7_group	0.0035	0.0009	0.1129	0.2919	0.3494	0.0879
Blautia	0.0081	0.0039	0.0619	0.1960	0.8060	0.3923
Coprococcus_2	0.0012	0.0043	0.0519	0.1721	0.1230	0.4276
Ruminococcaceae_UCG-014	0.0005	0.0026	0.2238	0.4697	0.0476	0.2559
Others					12.3011	8.7739

Euthyroid thyroid cancer D and Control C, (P < 0.05)

**Table 7:** High-throughput differential intestinal bacterial phylotypes between euthyroid thyroid cancer D and Control C at the species level

Taxa	Mean C	Mean D	p value	q value
Escherichia coli	0.026521	0.194062	0.016983	0.079437
Bacteroides vulgatus	0.166316	0.033518	0.004995	0.03812
Klebsiella_pneumoniae	0.03508	0.003094	0.022977	0.104115
Dorea longicatena	0.003479	0.035789	0.000999	0.013169
Bacteroides stercoris	0.065741	0.005255	0.001998	0.019314
Bacteroides uniformis	0.028511	0.006969	0.00999	0.060356
Akkermansia muciniphila	0.000379	0.01261	0.001998	0.019314
Parabacteroides distasonis	0.018982	0.004001	0.015984	0.079437
Bacteroides cellulosilyticus	0.004009	0.015244	0.032967	0.119505
Dialistersuccinatiphilus	0.003622	0.000106	0.042957	0.144855
Bacteroides coprophilus;	0.0039	0.000248	0.008991	0.059259
Bacteroides plebeius	0.006049	0.000388	0.002997	0.025563
Bacteroides massiliensis	0.006224	0.000758	0.002997	0.025563
Roseburia inulinivorans;	0.010472	0.002868	0.001998	0.019314
Bacteroides thetaiotaomicron;	0.007045	0.002454	0.026973	0.111745
Subdoligranulum_sp._4_3_54A2FAA	0.00044	0.002646	0.001998	0.019314
Lactobacillus mucosae	0.000586	1.87E-05	0.030969	0.115141
Bacteroides salyersiae	0.000504	3.17E-05	0.015984	0.079437
Fusobacterium varium;	0.00078	2.43E-05	0.030969	0.115141
Bacteroides eggerthii	0.000621	8.40E-05	0.02997	0.115141
Dorea formicigenerans	0.000351	0.001935	0.000999	0.013169
[Clostridium] leptum	6.91E-05	0.000674	0.090439	0.113169
Faecalibacterium_prausnitzii	7.09E-05	0.001133	0.00999	0.013169
Lactobacillusruminis	0.000215	1.87E-06	0.011988	0.06953
Ruminococcus_sp._16442;	1.49E-05	0.000155	0.023976	0.105349
[Clostridium] scindens	0.000101	0.000317	0.030969	0.115141
Paraprevotella xylaniphila;	0.000106	3.73E-06	0.016983	0.079437
Pseudomonas caeni	0.000105	3.73E-06	0.008991	0.059259
Pyramidobacter piscolens	0	9.52E-05	0.000999	0.013169
Acinetobacter_lwoffii	3.73E-06	8.21E-05	0.026973	0.111745

P<0.05.
