# Supplementary material for: Gut-Thyroid axis: How gut microbial dysbiosis associated with euthyroid thyroid cancer
Source: J Cancer. 2022 Mar 28;13(6):2014–28. doi: 10.7150/jca.66816 (PMC8990431; doi:10.7150/jca.66816)
Supplement: Supplementary file 1 — Supplementary tables. [file jcav13p2014s1.pdf]

Supplementary Information

Table S1. Serum thyroid antibodies and hormone in euthyroid thyroid cancer patients.

Patients	Age/Gender	T3 (ng/ml)	T4 (µg/dl)	FT3 (pmol/L)	FT4 (pmol/L)	TSH (µIU/ml)	Anti-TPO (U/ml)	Anti-TG Ab(%)	TM-Ab (IU/L)	Type of Thyroid Cancer
1	38/Female	0.85	8.09	5.19	15.90	2.81	<15.0	1.22	2.21	Papillary Thyroid cancer
2	35/Female	1.25	8.23	4.67	11.88	2.07	<15.0	4.06	1.10	Papillary Thyroid cancer
3	48/Male	0.95	5.90	4.30	12.70	1.90	<15.0	3.03	2.70	Papillary Thyroid cancer
4	50/Female	0.92	7.30	6.26	13.12	3.20	12	2.30	3.40	Papillary Thyroid cancer
5	40/Male	0.83	7.40	3.70	12.30	1.80	10	3.40	1.92	Papillary Thyroid cancer
6	30/Male	0.97	6.70	5.15	11.10	1.70	8	4.20	2.20	Papillary Thyroid cancer
7	43/Female	1.30	7.70	4.40	13.20	1.94	7	2.70	1.75	Papillary Thyroid cancer
8	46/Female	0.97	6.30	6.10	12.50	2.60	9	3.20	1.60	Papillary Thyroid cancer
9	49/Male	1.20	8.20	4.13	14.50	1.97	6	2.90	1.70	Papillary Thyroid cancer
10	33/Female	0.97	7.30	3.90	11.70	1.95	11	3.70	1.60	Follicular Thyroid cancer
11	42/Female	0.94	8.30	3.95	11.30	2.09	9	1.70	1.90	Papillary Thyroid cancer
12	36/Male	0.95	7.90	5.30	13.70	2.10	6	1.80	1.40	Papillary Thyroid cancer
13	31/Female	1.10	8.06	5.10	12.10	2.32	7	3.60	2.10	Follicular Thyroid cancer
14	47/Male	1.11	6.94	3.40	12.30	2.30	7	1.80	2.11	Follicular Thyroid cancer
15	39/Male	1.12	7.45	3.90	14.30	2.23	5	2.55	1.96	Papillary Thyroid cancer
16	37/ Male	0.94	8.07	4.23	11.70	1.98	6	2.10	1.30	Papillary Thyroid cancer

Table S2. Serum thyroid antibodies and hormone in healthy subjects.

**Control**	**Age/Gender**	**T3 (ng/ml)**	**T4 (μg/dL)**	**FT3 (pmol/L)**	**FT4 (pmol/L)**	**TSH (μIU/ml)**	**Anti-TPO (U/ml)**	**Anti-TG Ab(%)**	**TM-Ab (IU/L)**
1	35/Female	1.09	7.11	4.14	12.1	1.96	Negative	Negative	Negative
2	41/Female	0.99	5.31	3.67	13.31	1.21	Negative	Negative	Negative
3	40/Male	1.08	4.21	3.88	14.23	1.86	Negative	Negative	Negative
4	36/Male	0.91	4.32	3.63	9.19	2.12	Negative	Negative	Negative
5	38/Male	0.94	6.12	5.11	15.12	1.37	Negative	Negative	Negative
6	49/Female	1.13	5.61	4.23	11.13	1.98	Negative	Negative	Negative
7	50/Male	0.91	5.82	5.15	10.16	3.13	Negative	Negative	Negative
8	45/Female	0.89	6.42	4.14	14.17	2.51	Negative	Negative	Negative
9	37/Female	0.88	4.65	3.67	13.19	3.23	Negative	Negative	Negative
10	43/ Male	0.92	5.71	6.18	11.41	2.45	Negative	Negative	Negative
